# Cognitive Assessment of Children Who Are Deafblind: Perspectives and Suggestions for Assessments

**DOI:** 10.3389/fpsyg.2020.571358

**Published:** 2020-09-25

**Authors:** Jude Nicholas

**Affiliations:** ^1^Haukeland University Hospital, Bergen, Norway; ^2^Statped, Bergen, Norway

**Keywords:** cognitive potentials, communicative challenges, reliable assessments, assessment concessions, multiple assessment pathways

## Abstract

The overall goal of a cognitive assessment is to improve communication, learning, and quality of life for a child who is deafblind. This article will give a brief description and perspective on different evaluation approaches as a basis for reliable cognitive assessments and offer suggestions on how to improve the quality of a cognitive assessment in our clinical practice. The assessor should be aware of the limitations of norm-referenced tests if standardized normative measures are applied to evaluate the cognitive functions of a child who is deafblind. However, if engaging a child with deafblindness in a standardized normative assessment, special considerations and assessment concessions would be required. Furthermore, key issues on how to improve the quality of a cognitive assessment by affording multiple assessment pathways for cognitive assessments will be addressed. Particular attention is given to the following assessment approaches: multi-method, multi-informant assessment, ecological assessment, and dynamic assessment. The use of multiple assessment pathways is necessary to reveal the genuine cognitive abilities and potentials of a child with deafblindness.

## Introduction

The aim of any cognitive assessment in children is to evaluate cognitive functioning, to distinguish clinically abnormal from normal cognitive changes, and to provide information for determining appropriate goals and interventions. However, the cognitive assessment of children with deafblindness is not a straightforward process. Although accurate cognitive assessment of children with hearing impairment or visual impairment has been standardized, cognitive assessment of individuals with dual sensory impairment is more elusive (Hartshorne and Salem-Hartshorne, 2011). Children who are deafblind comprise a heterogeneous group. They are diverse in their sensory impairments, cognitive capabilities, and vulnerabilities. Notably, children who are deafblind “differ by type and level of hearing and vision loss, age of onset of vision and hearing loss, physical and health issues, cognitive functioning, expressive and receptive communication forms, and educational histories” (Bruce et al., 2018, p. 80). Besides, factors related to the etiology of deafblindness and co-existing neurological conditions associated with or completely independent from the etiology (i.e., epileptic seizures, cranial nerve problems, motor impairments, brain malformations, and brain related processing disorders), jointly may influence cognitive performance.

A child who is deafblind “face greater demands compared to sighted and hearing children in understanding how to relate to the surrounding world” (Bruce et al., 2018, p. 85). Access to the environment is limited in a child who is deafblind, which results in fragmented experiences and reduced learning opportunities. Children who are deafblind have unique communicative challenges due to their concurrent hearing and vision impairment. Both communicational and cognitive processes overlap. “Understanding the world and one’s relationship to it, the essence of cognition, is not easily separated from the child’s communicative abilities” (Rowland, 2009, p. 5). Accordingly, a child who is deafblind faces unique challenges that may affect many aspects of his/her inherent capacity that are needed for cognitive development. Consequently, cognitive assessments of a child who is deafblind must carefully consider how the child’s concomitant hearing and visual impairments and communicative abilities could affect a child’s cognitive performance.

Cognitive assessment of children who are deafblind is a challenge to all concerned. Children who are deafblind have multiple barriers to learning and information gathering due to their combined hearing-vision loss, which may mask their cognitive abilities. While there are “established guidelines and options for conducting cognitive assessment with individuals with visual impairment/blindness and hearing impairment/deafness” (Hill-Briggs et al., 2007, p. 389), practice suggestions for cognitive assessment in individuals with deafblindness are very limited.

## Different Evaluation Approaches as a Basis for Reliable Cognitive Assessments

### Standardized Normative Assessment

Standardized normative assessments “typically involve direct testing of an individual with a set of tasks administered under standardized conditions that permit comparisons to norms or to absolute standards for performance” [National Research Council (NRC) Committee on Disability Determination for Mental Retardation, 2002, p. 95]. The gold standard of cognitive assessing is “a face-to-face administration of a battery of standardized tests concerned with the objective measurement of cognitive abilities” (Herr and Ankri, 2013, p. 47). A variety of standardized normative tests has been published to assess cognitive abilities in children. For a further description and a list of the frequently used evidence-based cognitive or neuropsychological measures for children, see Campbell et al. (2008).

Some of these evidence-based cognitive/neuropsychological measures (nonverbal cognitive ability/attention and executive functioning/memory and learning) have been administered to children with deafblindness who display various degrees of visual and auditory impairment. For example, (a) the Wechsler Nonverbal Scale of Ability (Wechsler and Naglieri, 2006) has been administered to children with hearing loss and visual disability (Cupples et al., 2018); (b) subtests from the developmental neuropsychological assessment (NEPSY; Korkman et al., 1998) that evaluates planning skills (tower test) selecting and matching target visual stimuli (visual attention), memorizing and putting in order a story (narrative memory test) and the Rey’s Figure copying test (Rey and Osterrieth, 1993) that measures visuospatial construction and short-term visual retention have been administered in children with CHARGE syndrome with a wide range of visual and auditory deficits (Lasserre et al., 2013).

However, numerous challenges confront the assessor when using standardized normative measures to assess domain general or domain specific cognitive abilities in children with deafblindness. Generally, the assessor may lack knowledge of the nature of deafblindness, additional medical conditions may be present, and the assessment might be quite extensive and involves considerable time to both plan and carry out. More specifically, communication challenges exist. The communication of a child who is deafblind may be unconventional, prelinguistic, or non-symbolic (i.e., gestures and vocalizations). Many children with deafblindness may not be intentional in their communication with others (Damen et al., 2015). Moreover, their communicative expressions are subtle, their communicative attempts may be easily overlooked, and their responses may be difficult to judge during the assessment.

Furthermore, “most standardized measures are inappropriate because they have been normed for children with normal vision and hearing and they scarcely include children who are deafblind as a norming group” (Chen et al., 2009, p. 328). Most evidence-based cognitive/neuropsychological measures rely on use of vision and hearing and include auditory-verbal and visual-nonverbal tests which pose extreme barriers to gaining accurate information about the cognitive functions of a child who is deafblind. Valid assessment of children with deafblindness requires tools appropriate to this population. No valid and reliable standardized normative cognitive measures exist for this target group, across age or the deafblindness spectrum. The low-incidence nature of deafblindness and the heterogeneity among children with deafblindness makes it difficult to obtain sufficiently large sample sizes to develop valid normative data for cognitive tests.

The assessor should understand that the use of formal cognitive tests to obtain psychometric data in a more standardized manner may not be appropriate, when assessing a child who is deafblind. “Standardized instruments require precise administration procedures that may not allow enough flexibility to accommodate the needs of children who are deafblind” (Bruce et al., 2018, p. 85). However, if standardized normative cognitive tests are used it should consider the communicative challenges, psychosocial issues, and support needs of the individual child. Besides, it has been recommended that “if direct assessment is conducted it should be by or in the presence of at least one adult who knows the child with deafblindness well” (Nelson et al., 2002, p. 103). A standardized cognitive assessment may be helpful to identify the child’s cognitive capabilities and vulnerabilities in relation to their learning, as it may provide a snapshot of how a child with deafblindness is learning at that time in a controlled way. Consequently, “when used wisely and in consideration of each test’s limitations, carefully selected standardized tests may provide valuable information about the deafblind student’s development” (Wolford, 2016, p. 10). Furthermore, a cognitive profile of a child who is deafblind obtained by standardized assessments may aid in understanding the complex issues regarding differential diagnosis and the co-occurrence of neurodevelopmental disorders in deafblindness. Although standardized tools could be applied, expert judgment by professionals who have sufficient knowledge of assessing children with dual sensory/clinical experience with children having neurodevelopmental disorders should be required in a differential diagnostic process (i.e., deafblindness and intellectual disability). It has been described that “clinical experience is needed, as well as cross-disciplinary cooperation and specialized diagnostic methods together with an observation and intervention period in order to be able to assess and differentiate mental and behavioral symptoms from sensory deprivation in people with congenital deafblindness” (Dammeyer, 2011, p. 571).

Standardized normative measures can be helpful, but they must be used cautiously, especially when attempting to report an actual score. Typical scores used with norm-referenced tests (i.e., percentiles, standard scores, and T-scores) may not be reliable or valid once they deviate from standardized procedures; “they may do not correspond to the deafblind child’s actual capabilities, educational priorities, and learning experiences and they may underestimate or overestimate the child’s true potential” (Mar, 1998, p. 5). For this reason, when using standardized normative cognitive measures to evaluate a child who is deafblind, there is risk that the test scores may lead to incorrect judgments about the child’s cognitive functions, subsequently the assessor is at risk of giving wrong labels and diagnostic overshadowing may occur. Nevertheless, if engaging a child with deafblindness in a standardized normative cognitive assessment, special considerations may be required. Furthermore, the assessor should consider applying specialized measures or permitting assessment concessions to evidence-based tests.

Due to the loss of vision and hearing, the child who is deafblind may be better equipped to display his/her cognitive abilities through the act of touching (i.e., active touch). “Active touch or haptics refers to the act of touching, implies voluntary, self-generated movements and allows the individual to tactilely manipulate objects in trying to identify or remember them” (Nicholas et al., 2019, p. 24). Active touch/haptics may portray as a possible sensory modality to measure cognitive abilities in children with deafblindness. There is evidence for an empirical relationship between tactile measures and cognitive ability (Decker, 2010). Specialized measures, where active touch is used to assess specific cognitive functions may perhaps provide us with a better understanding of the child’s abilities. Some psychometric tactile cognitive measures with no reliance on vision (i.e., tactile measures in the Halstead-Reitan Test Battery: Reitan and Wolfson, 1993; tactile measures in the Dean-Woodcock Test Battery: Dean and Woodcock, 2003; and Haptic Test Battery: Ballesteros et al., 2005) have been administered to visually impaired children (Mazella et al., 2014). Unfortunately, these tactile measures lack sufficient or appropriate norms for children with deafblindness. Besides, tactile test performances that require the manipulation of haptic stimuli may be confounded by poor tactile/proprioceptive function or fine/gross motor impairments in some children with deafblindness. However, when administrating standardized tactile cognitive measures in the evaluation of a child who is deafblind, it is crucial that the results of these tests be interpreted within a broader context, including test norms, impact of level of visual, auditory and motor functioning and the child’s experience of everyday interactions with objects. Particularly, a functional assessment on how the child uses his/her sense of touch should be determined. Observational instruments designed to assess object manipulation skills could be useful, for example, the Home Inventory of Problem Solving Skills/School Inventory of Problem Solving Skills are observational instruments designed to assess skills related to object use in children who are deafblind or have severe and multiple disabilities (see Rowland, 2009).

Since children with deafblindness often do not respond in a standard manner to formal tests and they may score at floor levels for many of the tasks, it is important that evaluators consider flexible procedures when evaluating a child who is deafblind. For this reason, when engaging a child with deafblindness in a standardized cognitive assessment, special considerations such as assessment concession would be required.

Assessment concession “revolves around an effort to minimize the impact of a range of intrinsic and extrinsic barriers upon the assessment performance of the learner” (Alant and Casey, 2005, p. 186). Assessment concessions for a child who is deafblind are an intricate issue and the type of concessions made to cognitive assessments varies. Two categories of assessment concessions will be described: test accommodation and test modification.

Test accommodation concerns potential changes in the ways assessment tasks are presented to the instructional needs of the child. Test accommodations “improve accessibility to the content of the test and are selected to remove barriers that inhibit the learner’s ability to demonstrate knowledge” (Cawthon, 2010, p. 192). Generally, children with impairments benefit from test accommodations, “but for some children, the accommodated instrument would appear to be unsuitable because the impairment could be too severe” (Visser et al., 2013, p. 3741). Nevertheless, in the case of deafblindness, accommodation is about how to adjust the cognitive assessment in a personalized way, so that the impact of visual and hearing difficulties on test performance are reduced, while not affecting what the test measures.

While many of the test accommodation principles described here are applicable to cognitive assessment of children with special needs in general, some of these test accommodations are particularly relevant for children with deafblindness. Some examples of test accommodation for children with deafblindness are the following:

- Presentation accommodations (to change how assessment information and instructions are presented): enlarging, brightening, or changing the contrast of test items; using sound amplification devices; providing visual, auditory, and touch cues to support attention and engagement; and providing appropriate communication mode for test instructions and content (i.e., hands-on methods of demonstration and instructions, having a sign language interpreter delivering instructions, using visual or tactile/haptic signs).
- Response accommodations (to allow test responses to be given through ways other than typical verbal or written response): allowing for basic forms of communication (i.e., gestures or vocalizations); allowing for some form of pointing (i.e., finger and hand pointing, eye gazing, or visual scanning); allowing for using aided communication devices (i.e., picture boards, tablet-based technology, speech generating devices, or a brailler); and allowing for using visual or tactile signs.
- Setting accommodations (to change how the environment is structured): reducing visual and auditory distractions in the test room (i.e., reduction of visual clutter and limiting background noise); room design modifications (i.e., specialized lighting and noise reduction materials); enhancing speech reading conditions (i.e., avoiding hands in front of face, clearly enunciating speech); and providing physically accessible resources (i.e., seating in close proximity to the assessor, adaptive furniture, or equipment).
- Timing and scheduling accommodations (to change how time is organized): providing extra practice trails; providing frequent breaks and multiple sessions to complete tests; allowing extra time for processing auditory information (i.e., repeating/rephrasing information when necessary); allowing extra time for processing visual information (i.e., extended time for critical visual details when completing visual-perceptual tests); and allowing flexibility according to the child’s physical states (i.e., motor impairments, level of reduced energy/fatigue due to medical conditions or medications, level of physiological arousal/pain) or psychological states (i.e., disinterest, lack of motivation, feelings of difficulty or frustration, and level of stress/anxiety). Hence, it is important to evaluate the child’s physiological and psychological states during the assessment in this regard. For example, an observational and qualitative analysis scale has been developed to evaluate a child’s behavior emotional, behavioral and attention abilities during the testing session (see Löfkvist et al., 2020).

In contrast to test accommodation, test modification is considered “as a change in the nature or content of the assessment” (Conderman et al., 2017, p. 72). Modification is described as “an alternate assessment and content changes may significantly alter the context and complexity level of the assessment” (Alant and Casey, 2005, p. 188). Furthermore, the assessor must remain aware “that by virtue of using test modifications, validity of normative data and interpretation guidelines may be breached” (Hill-Briggs et al., 2007, p. 401). Examples of test modification for children with deafblindness would imply modifying mainstream tests/subtests, such as transforming visual test items to manually explorable/tangible representations (i.e., into three-dimensional representations), replacing visual-spatial test materials with tactile/haptic versions (i.e., textured and raised materials) or translating auditory-verbal test items into equivalent visual or tactile signs.

It is likely that potential difficulties may emerge when differentiating between accommodations and modifications. In the case of deafblindness, test item translation into visual or tactile signs is one such example. “Test translations traditionally can be regarded as an accommodation, provided that the content of the test is not changed” (Alant and Casey, 2005, p. 187). However, literal translations of tests are difficult and not always possible, granting that test modifications need to be considered. For example, the translation of auditory-verbal tests [i.e., verbal comprehension subtests in the Wechsler Intelligence Scale for Children-Fifth Editon (WISC-V): Wechsler, 2014] into equivalent visual signs may significantly modify items and alter the task demand. “Individual test items, when translated into visual sign, may be comparatively more or less difficult than the original English item” (Day et al., 2015).

Furthermore, when administering an auditory-verbal working memory test (i.e., Digit Span subtest in the WISC-V; Wechsler, 2014) “in a signed language modifies the cognitive demands of this subtest from a task that taps auditory working memory to one that taps visual working memory and this modification significantly alters the construct being measured” (Day et al., 2015, p. 7). Similarly, when administering a visual processing speed test (i.e., Symbol Search subtest, in the WISC-V; Wechsler, 2014) that allows for extended time to complete the test may alter the speed construct being measured.

Although clinical judgment and experience have led to some recommended assessment concessions practices for children with deafblindness, limited research has been done to prove the validity of test modifications that depart from standardized test procedures (i.e., test items presented in a tactual form, test items translated into visual/tactile sign or extended time on visual tasks). Although, a study has evaluated the validity of transforming mainstream visual and auditory-based tests into the tactile modality in adults with dual sensory loss (Bruhn and Dammeyer, 2018), there are no valid and reliable transformed tactile cognitive tests which could be applied to children with significant vision and hearing loss. Although test modification practices could reveal the true potential of a child with deafblindness, the assessor must be extremely cautious when interpreting test results or explaining test scores when test modifications have been applied. It has been suggested that “great caution should be applied when estimating the abilities of children who are deafblind” (Geenens, 1999, p. 162).

Assessment concessions may be appropriate only if the assessment is based on a clearly articulated purpose and the cognitive measures are selected to address the types of functions that harmonize with the child’s learning experiences and intervention goals. Subsequently, for a child who is deafblind to be meaningfully evaluated, the assessment procedure should be highly individualized and the cognitive measures must be carefully chosen and planned to answer questions unique to each child’s learning needs or functional life skills. For this specific purpose, a qualitative evaluative approach that involves evaluating a child’s cognitive capabilities that reveal appropriate interventions may yield a more valid assessment when evaluating a child who is deafblind. Accordingly, the cognitive/neuropsychological items should be selected and guided to identify the basic and complex cognitive concerns from a child’s on-task behavior, as shown in these examples: how information is best communicated to promote learning; how does perceptual-cognitive characteristics in the different modalities help promote the child’s multisensory learning; how does the child’s spatial ability in the visual or tactile modality affects his/her explorative activity, navigation, or mobility training (spatial cognition); how does the child stays focused and problem solve when a new or unfamiliar task is presented (attentional switching); how many repetitions of information does it take for the child to acquire new information (working memory); how does the child learn to remember specific personal episodes/experiences (autobiographical memory); how does the child’s ability to generate a plan affects his/her functions in everyday life (cognitive planning); how effectively does the child use feedback from the environment (cognitive flexibility); how does the child allow for change in perspectives in order to reach a goal (goal-oriented); and what is the child aware of when facing a task and processing the information related to it (metacognitive knowledge). A qualitative evaluative approach to cognitive assessment may reveal what the child needs regarding learning and daily life functioning and that “the most important assessment goal is to gain an understanding of the child’s real-life skills and concepts as applied in educational, home, and social settings” (Chen et al., 2009, p. 332).

As parents and educators become more aware of the barriers of standardized normative cognitive assessments, a discussion on the implications of assessment concessions tends to become necessary. Nevertheless, cognitive assessments of children with deafblindness should focus on the processes that promote learning, problem solving, and functional skills rather than on the norm-referenced test scores. In other words, a qualitative approach to cognitive assessment may give children with deafblindness the opportunity to reveal their genuine cognitive abilities and potentials. A study has described that when the quality of interaction was optimized and the individual assessment concessions regarding visual and auditory limitations were applied to the Bayley Scales III (Bayley, 2006), the qualitative assessment was able to capture latent cognitive processes and reveal critical functional skills (i.e., problem-solving ability) in persons with congenital deafblindness (Tuomi, 2014).

Furthermore, rating instruments/questionnaires may be suitable for assessing cognitive abilities and have demonstrated their usefulness as a supplement to standardized measures (Gioia et al., 2000). Standardized tests may “deconstruct cognitive function precisely but may lack the ability to predict behaviors in real-world settings” (Egeland et al., 2017, p. 231); conversely, rating instruments/questionnaires are designed to capture behavior in real-life situations. Rating measures are useful in collecting and quantifying observed behavior. There are several rating instruments available to assess cognitive abilities in children, some examples include: (a) the ability to carry out tasks of daily living/adaptive behavior [i.e., Vineland Adaptive Behavior Scales (Vineland-3); Sparrow et al., 2016 and Adaptive Behavior Evaluation Scale (ABES-3); Harrison and Oakland, 2015] and (b) the ability to manage oneself in flexible ways/executive functions [i.e., Behavior Rating Inventory of Executive Function (BRIEF); Gioia et al., 2000]. The Vineland, ABES, and BRIEF have been used to assess cognitive abilities in children with CHARGE syndrome (Salem-Hartshorne and Jacob, 2005; Hartshorne et al., 2007; Abadie et al., 2020).

It is reasonable to say that there are almost no standardized rating instruments that include specific norms for comparisons with children who are deafblind. Consequently, adaptive behavior scales “are not especially sensitive to the development and learning modalities of children who are deafblind” (Chen et al., 2009, p. 326). Thus, the evaluator must be aware that some items or domains in traditional rating measures are probably inappropriate and could be easily misinterpreted (Salem-Hartshorne and Jacob, 2005). Accordingly, the overall scale profile would appear atypical and might not cover the scope of the functional skills that a child who is deafblind has achieved. Hence, when an adaptive behavior scale is applied, it is important that information about the child’s functional ability is gathered from multiple sources and then integrated with the results from the behavior scale to make important decisions about the overall cognitive functioning of a child who is deafblind.

However, there are a few rating measures designed specifically for children with deafblindness, for example, the Callier-Azusa Scale (Stillman, 1974) and the Child-Guided Strategies (Nelson et al., 2002). A case study has shown that when using the Child-Guided Strategies as an assessment measure, it was possible to reveal fundamental problem solving and memory skills that provided information for further support for a child with deafblindness (Damen, 2020).

In essence, the use of standardized normative measures alone is insufficient to yield accurate predictions of cognitive abilities in children with deafblindness. It is, therefore, essential that a child who is deafblind be afforded multiple assessment pathways for cognitive assessments.

### Multi-Method, Multi-Informant Assessments

The use of a multi-method, multi-informant assessments are necessary given the limitations of available standardized normative measures. Since children who are deafblind comprise a heterogeneous group and are diverse in their cognitive capabilities, an individualized approach is required. An individualized assessment begins with understanding the sensory functions of a child who is deafblind. “Assessment of children who are deafblind should include functional vision and hearing evaluations to augment information from the audiology and ophthalmology reports” (Bruce et al., 2018, p. 86). Besides, the functional vision and hearing assessment must include assessment of brain related visual and hearing loss (i.e., CVI and APD) when suspected (Nicholas et al., 2019). The term brain related visual and hearing loss is used “when a neurological impairment is affecting the normal functioning of vision and hearing, due to central damage to the visual and auditory processing areas in the brain” (Saunders and Echt, 2012, p. 1044). In these cases, a combination of procedures is necessary to discover each child’s unique cognitive abilities.

Best practices in cognitive assessment call for a multi-method, multi-informant assessment approach which highlights the importance of gathering information from several sources to improve the accuracy and quality of the cognitive assessment. For example, multi-method, multi-informant assessments would include gathering comprehensive information through previous reports (medical or developmental findings), informal observations, checklists, and interviews with teachers, other professionals, caregivers, and family members to understand the full scope of the child’s cognitive capabilities and vulnerabilities.

Accordingly, due to the complexity and heterogeneity of deafblindness, it is important that a multidisciplinary approach using interprofessional collaboration be established when conducting a cognitive evaluation of a child who is deafblind. An interprofessional collaboration using a multidisciplinary approach is described as “having different professions coming together to work toward a common goal and that all team members embrace the unique perspectives of all other team members” (Strunk et al., 2017, p. 61). By working together team members would be able to identify informational needs, gather necessary information and assess areas of concern through observation or tests.

A multidisciplinary approach to cognitive assessment in which professionals adopt a truly interprofessional collaboration built on shared goals and shared knowledge should also include the family member’s description of the child’s resources, priorities, and concerns. The active inclusion of family members during the cognitive evaluation is essential, since they can provide information regarding the child’s cognitive behavior in the home and within other environments. Both parents as informants can provide different perspectives and insights about their child who is deafblind that “might not be observed within the limited time frame of an evaluation” (Mar, 1998, p. 5).

Furthermore, the outcomes of the cognitive assessment conducted through an interprofessional collaborative practice could be used to develop meaningful interventions and services, specifically family interventions. In particular, family members should be advised against having low expectations for a child who is deafblind and at the same time given a more pragmatic understanding of their child’s cognitive vulnerabilities.

### Ecological Assessment

A major consequence of only using standardized measures is that less focus is given to environments or systems that might influence the child’s cognitive competency in his/her everyday life. Contrarily, an ecological assessment, a non-standardized approach, may be better equipped to capture a child’s cognitive behaviors in different situations or settings. Accordingly, a neuropsychological assessment conceptualized within an ecological neuropsychology perspective, rather than within the traditional deficit model, emphasizes a “strength-based approach that considers the child, as well as the systems within which he/she interacts, when assessing, diagnosing, and intervening with students who are experiencing learning difficulties” (D’Amato et al., 2005, p. 97).

An ecological assessment focusses on recognizing environments or settings in which the child with deafblindness will function on an optimal level. The assessment implies conducting structured observations in multiple settings. The assessment process begins with identifying certain cognitive domains of the child and the emergent environments in which the child presently functions. An ecological assessment would involve several steps. O’Reilly et al. (2007, p. 148) describe an ecological assessment in a five-step process: “(1) identify the core performance domains, (2) identify the environments in each of the domains, (3) divide the environments into sub-environments, (4) identify the critical activities within each sub-environments, and (5) assess the child’s performance on each of the critical skills.” Importantly, parents and teachers/staff should be involved when using an ecological assessment approach. Indeed, in an ecological neuropsychology assessment understanding how the home and other environments “may have to change to accommodate the child’s needs should be considered” (D’Amato et al., 2005, p. 97).

An ecological assessment is a broad scope assessment that is useful to gather information about how a child functions in various environments and in interaction with different adults. It has been reported that “children who are deafblind function differently across environments” (Bruce et al., 2018, p. 85). Consequently, in an ecological assessment approach “effective assessments are conducted across multiple and natural environments with input from multiple adults” (Chen et al., 2009, p. 329).

The essence of assessing cognitions applying an ecological approach is to capture subtle behavioral components from naturalistic environments by empirical observation that corresponds to the child’s unique cognitive functions. In other words, an ecological assessment “demonstrates how intra-individual characteristics interact with other aspects of the learning environment to produce a more ecologically oriented picture of the child’s cognitive capabilities” (D’Amato et al., 2005, p. 101). A case study has shown that by identifying the behavioral components that indicate cognitive functions (i.e., executive functions and spatial cognition) in an environment that motivated the child with deafblindness to be engaged in (i.e., indoor climbing activity), it was possible to detect and describe the targeted cognitive abilities needed to perform the function (Gibson et al., 2020). Consequently, this indicates that an ecological assessment can be helpful to identify cognitive functions in environments that allow a child who is deafblind to participate with proper support. It is important to be aware that not only the physical environment (i.e., critical components of the learning environment) but also the social environment (i.e., the quality of the interactions, communication competence of the partner) has influence on the outcome of the cognitive assessment. “It is essential to focus on the competences of the communication partners, when assessing the potentials of people with congenital deafblindness” (Boers et al., 2013, p. 129). Individuals with deafblindness can display surprising cognitive skills in proficient interaction with a competent communication partner, “and these skills can develop when interaction is improved” (Ask Larsen and Damen, 2014, p. 32).

An ecological based cognitive assessment is a function-led approach that implies that the observation of cognitive behaviors could be done by direct observation and by video observation. Since the communicative expressions of children with deafblindness are subtle, easily overlooked and difficult to judge, it is recommended that video use recordings during assessment to capture the subtle behavioral cues. It can be assumed that these “subtle behavioral cues could have been missed when direct observation methods are used, since the bodily signals or emotional expressions of children with deafblindness are often subtle, can unfold at slow pace, pass unnoticed, and can be difficult to interpret or read” (Nicholas et al., 2019, p. 62). The use of video observation has become part of the standard procedure when assessing the dyadic interaction between the individual with deafblindness and the communicative partner (Janssen et al., 2007b; Dammeyer, 2012; Damen et al., 2014). However, through observations from video recordings, it will be also possible for the assessor to notice subtle behavioral cues and analyze the child’s behavior in a very minute way in order to reveal crucial aspects of the child’s cognitive abilities.

Moreover, video-feedback intervention, “a well-known instructional method that is applied in different training programs in order to improve the interaction skills of professionals” (Fukkink et al., 2011, p. 56), has been demonstrated to improve interactions and communication between individuals with congenital deafblindness their communication partners (Damen et al., 2020). Although video-feedback interventions have traditionally focused on the aspects of communication, it is suggested that video-feedback interventions should also be applied to stimulate more basic and complex forms of cognitive functions, for example, by scaffolding individualized cognitive strategies during interactions between a child who is deafblind and his/her communication partner.

### Dynamic Assessment

Dynamic assessment is an assessment model that “integrates assessment and instruction into a seamless, unified activity aimed at promoting learner development through appropriate forms of mediation that are sensitive to the individual’s current abilities” (Lantolf and Poehner, 2004, p. 50). The essence of dynamic assessment is mediated assistance and by performing cognitive assessment in a dynamic manner, the assessor may get a better opportunity to obtain an insight into the child’s cognitive potentials. “Dynamic assessment focuses on modifiability and on producing suggestions for interventions that appear successful in facilitating improved learner performance” (Lidz, 1991, p. 6).

The dynamic assessment model is influenced by two theoretical approaches. In the first theory, Vygotsky (1978, p. 201) highlights the concept of zone of proximal development and describes that “responsiveness to assistance is an indispensable feature for understanding cognitive ability because it provides an insight into the person’s future development.” In other words, “what the individual is able to do currently with assistance, he or she is able to do later alone” (Lantolf and Poehner, 2004, p. 51). In the second theory, Feuerstein et al. (1988, p. 204) highlight the concept of mediated learning experience and describe that “the examiner is required to interact flexibly with the individual examinee, negotiate the assistance and guidance that is required to modify the cognitive structure of the individual.” For Feuerstein et al. (1988, p. 199), the aim of dynamic assessment is “to undo the predictive value of the initial assessment by modifying functioning through the mediational process.” Nevertheless, both of these theoretical approaches emphasize the concept of mediation. “Mediation arises through joint engagement with a cognitive task at hand: The role of the mediator is to gauge the level of the student’s functioning and to reformulate the task in such a form that the student can master the task” (Grigorenko, 2009, p. 117).

Dynamic assessment is an “umbrella term used to describe a heterogeneous range of approaches whose core is in blending instruction into assessment” (Grigorenko, 2009, p. 112). Two primary approaches of dynamic assessment will be described: interventionist and interactionist.

To begin with, the interventionist approach is described as a high-structure dynamic assessment in which “focus is on helping individuals become more efficient in their learning” (Lunt, 1993, p. 164). One type of format within the interventionist approach is referred to by as the “sandwich” format (Sternberg and Grigorenko, 2002). The “sandwich” format primarily relies on a pretest/assess – training/intervention – posttest/reassess layout and consists of layers of both assessment and intervention. The initial pretest/assess phase defines baseline performance, followed by the training/intervention phase and the final posttest/reassess phase suggests performance as “indicators of learning potential” (Guthke, 1982, p. 309). Accordingly, standard test measures could be used in the pretest/assess phase and specific instructions (i.e., systematic instructions/graduated guidance) are given during the training/intervention phase. Furthermore, the “sandwich” format examines performance under different instructional conditions and performance scores may then be “used to group learners as high scorers (i.e., those with high pre-training scores, and who therefore do not manifest much improvement as a result of training), gainers (i.e., those whose scores showed marked improvement as result of training), and non-gainers (i.e., those who performed poorly on the pretest and did not profit from instruction)” (Lantolf and Poehner, 2004, p. 55). Notably, certain aspects of the interventionist approach correspond with the ecological neuropsychology perspective, where intervention “not only focuses on remediation and compensation skills for the child but also on the match between the child and his/her instructional environment” (D’Amato and Rothlisberg, 1996, p. 672).

Next, the interactionist approach is described as a low-structure dynamic assessment and allows the assessor “greater freedom to interact with the learner and thereby deploy a wide array of assistance to foster development” (Minick, 1987, p. 138). The interactionist approach points out that the “mediator not only modifies the stimuli or task but also affects the learner by arousing him or her to a higher level of curiosity and to a level at which structural cognitive changes can occur” (Sternberg and Grigorenko, 2002, p. 54).

Regardless that the interventionist or interactionist approach may differ in their procedures, both approaches “highlight the general principle that guided learning can make a valuable contribution to the assessment process” (Asha and Edvard, 1993, p. 10).

The interventionist approach to cognitive assessment has adapted test administration and has been applied to different groups of children with special educational needs to reveal learning potentials. For example, the interventionist approach has been put into practice in children with learning difficulties (Kirkwood et al., 2001), with visual impairment (Borca, 2013), and with deafness (Tzuriel and Caspi, 1992). The main reason for applying dynamic assessment with standardized tests is that “it allows more insight into the student’s learning potential than the test’s standard administration allows” (Wolford, 2016, p. 11).

Moreover, an interactionist approach to cognitive assessment that focused on a child diagnosed with ADHD and limited cognitive and linguistic abilities, demonstrated that through a dialogic interaction the child was able to learn to use her own speech for self-mediation, perform at an age-appropriate level and thereby overcome her challenges (Karpov and Gindis, 2000).

Generally, a dynamic assessment approach that highlights guided learning may provide a more accurate practice to reveal the genuine cognitive abilities and assist in identifying effective learning strategies in a child who is deafblind. Dynamic assessment procedures to measure the communication potential of people with congenital deafblindness have been investigated (Boers, 2015). However, little is known about the dynamic approach of cognitive assessment in children who are deafblind. A dynamic assessment approach (sandwich format) that uses test measures during the pretest phase and specific cognitive/metacognitive instructions during the training phase could provide useful information on whether a child who is deafblind would benefit from a cognitively-based structuring procedure. Accordingly, the recent work of Nicholas et al. (2019) link the assessment and intervention of working memory in the tactile modality through a dynamic assessment approach. Tactile working memory can be described as the ability to keep relevant tactile information in mind for a limited amount of time using active touch and is “involved in the storage and retrieval of information about objects that people explore using active touch and motion” (Gallace and Spence, 2009, p. 394). It has been suggested that children with deafblindness are highly capable in the use of active touch to get information (Janssen et al., 2007a), to learn (Silberman et al., 2004), and to develop personal memories (Gibson and Nicholas, 2017). The dynamic assessment of tactile working memory considers the optimization of the physical environment (i.e., the learning context), the social environment (i.e., partner competences), and the mediation of effective tactile learning strategies (i.e., perceptual, cognitive and social cognitive strategies) within the assessment. Moreover, this dynamic assessment procedure emphasizes a multi-informant and ecological neuropsychology approach (Nicholas et al., 2019).

In addition, a cognitive assessment should incorporate different aspects of social cognitive measures when assessing children who are deafblind. Many children with deafblindness may have difficulty in their social cognitive abilities due to dual sensory loss and communication challenges. Social cognition can be described as a complex social cognitive information process that is dependent on cognitive and socio-emotional aspects. Fundamentally, it is an ability to recognize emotion and emotion perception and that it “underlies capacities such as the ability to decode and understand social cues for making inferences about other people’s mental states (e.g., theory of mind reasoning), regulating emotions and feelings, and experiencing and expressing empathy” (Njomboro, 2017, p. 3). Moreover, the underlying capacities related to social cognitions are involved in the “know-how that allows us to sustain interactions, form relationships, understand each other, and act together” (De Jaegher et al., 2010, p. 441) and it is critical “for successful communication and, consequently, mental health and wellbeing” (Henry et al., 2016, p. 32). Although there are a few psychometric measures that evaluate social cognitive abilities in children (i.e., The Social Cognitive Evaluation Battery; Thiébaut et al., 2010), the social cognitive abilities of children with deafblindness are likely to be underestimated because the standardized procedures of such assessments are not appropriate for this heterogeneous group. For this reason, it is feasible that a multidisciplinary, ecological or dynamic approach to social cognitive assessment is adopted, with assessment and information regarding the child’s auditory and visual functions, communication skills and cognitive functions taken into account. Furthermore, it has been suggested a socio-cognitive framework may provide a useful framework “for teachers to support the symbolic understanding of school-aged children with deafblindness and understand their students’ individual socio-cognitive abilities and their social interactions” (Hartmann, 2012, p. 140).

## Summary

The purpose of this article is to provide a perspective on the different evaluation approaches as a basis for reliable cognitive assessments and offer suggestions on how to improve the quality of a cognitive assessment in children who are deafblind.

The overall goal of a cognitive assessment is to identify cognitive abilities and vulnerabilities, to assess their consequences in relation to learning and to obtain sufficient information for determining intervention priorities for each child. However, when assessing cognitions in child who is deafblind specific focus is needed on issues regarding test measurement, comorbid conditions as well as communication challenges. The assessor should be cautious when using standardized normative measures. However, if standardized tests are used the assessor should have a good knowledge of the nature of deafblindness and a good understanding of the implications of assessment concessions (i.e., accommodations and modifications). A valid assessment depends on the competence of the assessor evaluating the child who is deafblind and whether cooperation and attention of the child have been established. If standardized normative tools are used, it is necessary to make a detailed record of the type of assessment concessions applied. Interpretation of test results should be considered in the context of known challenges in using standardized normative cognitive tests in children with deafblindness. Consequently, typical test scores with norm-referenced measures must be used cautiously, especially when estimating the cognitive abilities of a child who is deafblind. The low-incidence nature of deafblindness and the heterogeneity among children with deafblindness makes it is difficult to obtain psychometrically-sound metrics of cognitive/neuropsychological functions in children who are deafblind. At present, there are no standardized normative cognitive measures for use specifically with this population and standardized normative tests present major limitations in terms of generalizability of behaviors in real life. In fact, a qualitative evaluative approach to cognitive assessment may yield a more valid assessment when evaluating a child who is deafblind. Hence, for the child with deafblindness to be meaningfully evaluated, the cognitive assessment must be planned and guided to identify the cognitive concerns from a child’s on-task behavior.

Since standardized normative parameters are less reliable and feasible, it is essential that a child who is deafblind is afforded multiple assessment pathways for cognitive assessments. The use of multiple assessment pathways is necessary to reveal genuine cognitive abilities because the sensory, communicational, and psychosocial concerns of a child with deafblindness are so complex. Multiple assessment pathways involve the use of multi-method, multi-informant assessment, ecological assessment, and dynamic assessment as a basis for reliable cognitive assessments and they may benefit the child who is deafblind to the greatest degree possible. In addition, multiple assessment pathways for cognitive assessment should also consider the evaluation of the different aspects of social cognitive abilities in children who are deafblind.

Since the communication expressions of a child who is deafblind are often subtle and difficult to judge, observations from video recordings are necessary to analyze the child’s behavior in a very minute way for the purpose of revealing the child’s cognitive abilities. Interprofessional collaboration using a multidisciplinary approach that includes family members may offer the most valid cognitive assessment for a child who is deafblind.

However, much remains to be understood about the cognitive processes involved in brain related visual and hearing loss in children who are deafblind. This requires further investigation.

The cognitive assessment of a child who is deafblind should be considered as a comprehensive evaluation process, in which the cognitive potentials and vulnerabilities are described in relation to the child’s visual, hearing, and tactile characteristics in order to ascertain pathways toward individualized interventions and support that could help the child, school staff and family members.

## Limitations

This article has potential methodological limitations that can impact the validity of the conclusions. A systematic search for peer reviewed journal articles was not carried out. Furthermore, a critical review of the literature on cognitive assessment for children with deafblindness was not presented in this article. Although there are a few studies that have provided a systemic literature search on this topic, the methodological limitations in this article could be addressed in future research. Nevertheless, this article presents a perspective on the different evaluation approaches as a basis for reliable cognitive assessments and offers research-based knowledge that informs effective cognitive assessment and intervention planning for children who are deafblind.

## Author Contributions

The author confirms being the sole contributor of this work and has approved it for publication.

### Conflict of Interest

The author declares that the research was conducted in the absence of any commercial or financial relationships that could be construed as a potential conflict of interest.
